# Factors Associated with Dietary Control and Physical Activity in the Management of Metabolic Syndrome in Korean Menopausal Women

**DOI:** 10.3390/ijerph17186901

**Published:** 2020-09-21

**Authors:** Guna Lee, Hye Young Choi

**Affiliations:** 1 Department of Nursing, Cheongju University, 298 Daesung-ro, Cheongwon-gu, Cheongju 28503, Korea; gunalee@cju.ac.kr; 2 Department of Nursing, Kangwon National University, 346, Hwangjo-gil, Dogye-eup, Samcheok-si, Gangwon-do 25949, Korea

**Keywords:** healthy diet, physical activity, metabolic syndrome, menopause, healthy lifestyle

## Abstract

The increased prevalence of metabolic syndrome (MetS) among menopausal women necessitates successful management strategies such as applying dietary restrictions and engaging in physical activity to improve their health and quality of life. We investigate factors associated with dietary control and physical activity in 564 menopausal Korean women classified as having MetS who partook in the 2016 and 2017 Korean National Health and Nutrition Examination Survey (KNHANES). To determine socio-demographic characteristics, lifestyle features, and MetS-related characteristics associated with dietary control and physical activity, multiple logistic regression analysis was performed. Of the women with MetS 36.1% applied diet control and 39.5% engaged in physical activity. Women who applied dietary control strategies to improve their health were more likely to be in the 40–49 age group (odds ratio (OR): 3.38; 95% confidence interval (CI): 1.25–9.18), to engage in physical activity (OR: 2.24; CI: 1.43–3.52), and to take hypertension medication (OR: 1.66; CI: 1.04–2.67) or diabetes mellitus medication (OR: 2.99; CI: 1.80–4.97). Physically active menopausal women with MetS were more likely to also engage in dieting (OR: 2.32; CI: 1.42–3.51). Accordingly, suggestions can be provided to healthcare workers in designing, not only individual approaches to lifestyle modification but also comprehensive interventions including dietary control and physical activity for menopausal MetS women. Health-care interventions like dietary control, which provide additional support to vulnerable MetS women, should target women aged 60 or above or those who do not take medicines for hypertension and diabetes mellitus.

## 1. Introduction

Menopause generally indicates the permanent cessation of ovulation and a decrease in estrogen and progesterone from decreased ovary activity. Estrogen deficiency occurs due from changes in body fat distribution and is associated with decreases in both vascular elasticity and endothelial cell function, as well as increases in low-density lipoprotein cholesterol [1]. Menopause is a risk factor for metabolic syndrome (MetS) as well as diabetes mellitus and cardiovascular diseases [2].

The global prevalence of MetS, which is a cluster of clinical features such as abdominal obesity, elevated triglycerides, blood pressure, plasma glucose, and reduced high-density lipoprotein (HDL) cholesterol [3], is increasing [4] and approximately 26–30% of Korean adults have MetS [5]. MetS has been associated with serious diseases, for example, diabetes, hypertension, hyperuricemia, cardiovascular diseases such as angina pectoris and myocardial infarction, stroke, and cancer, including breast cancer and colorectal cancer [6,7,8]. Various studies report that the prevalence of MetS in menopausal women is relatively higher than in premenopausal women [2,9]. Previous studies also report that MetS severity in menopausal women increases more rapidly than in pre-menopausal or peri-menopausal women [9]. This increased prevalence of MetS stimulates the necessity of MetS management in menopausal women for their health and quality of life.

Effectively managing MetS involves a combined approach of lifestyle modification and pharmacological interventions. Various studies suggest lifestyle modifications, including dietary control and physical activity for each body weight, to ensure successful MetS management [10,11]. Concentrated lifestyle modifications result in decreased weight, blood pressure, triglycerides, and fasting glucose levels and raising HDL cholesterol levels [12,13,14]. Dietary control and physical activity are especially important for lifestyle modifications to regulate all components of MetS [11,15].

Several studies state that MetS risk factors include age, race, dietary type, physical activities, and smoking [9,10,11,12]. However, there are insufficient data to prove the association between compliance with a healthy lifestyle and individual lifestyle characteristics or patterns [16,17]. Furthermore, factors associated with healthy lifestyle—including socio-demographic data and lifestyle features of menopausal MetS women—are nearly unknown. Therefore, in the current study, we aim to investigate factors, grouped by socio-economic status, lifestyle, and MetS status, associated with dietary control and physical activity in menopausal women with MetS, using a representative sample of Koreans.

## 2. Materials and Methods

### 2.1. Study Design

This secondary data analysis study, built on the Korean National Health and Nutrition Examination Survey (KNHANES), investigated MetS status and associated factors of dietary control and physical activities, targeting menopausal adult women with MetS.

### 2.2. Subject

This study used data from the seventh primary year (2016) and secondary year (2017) of KNHANES, conducted by the Korea Centers for Disease Control and Prevention (KCDC). The inclusion criteria for the study population were menopausal women aged 19–65 years who fall into the MetS category. The final 564 eligible people were selected by analysis from 16,277.

### 2.3. Data Collection

This study used subject-unidentifiable data in accordance with KNHANES’ data provision and processing procedures. The subject sample from the seventh KNHANES were chosen from the most recently conducted Population and Housing Census Survey (2010). This study used two-stage stratified cluster sampling and systematic sampling built on the survey’s boundary areas/household numbers to extract a representative sample.

This study used the following KNHANES’ factors: health survey, nutrition survey, and examination. The health survey and examination were conducted at a mobile examination center, and the nutrition survey was conducted by visiting the subjects’ house. The surveys were conducted using 1:1 interviews between the examiner and examinees, or by self-entry method, depending on the questionnaire’s details. The examination was performed with direct measurement, observation, and sample analysis. The examination team consisted of a nurse, nutritionist, public health specialist, and people who examined performance ability through level education, and on-site quality supervision.

To perform the secondary data analysis, institutional review board exemption approval was obtained from the affiliated university (Approved No. KWNUIRB-2020-01-010).

### 2.4. Variables

#### 2.4.1. Socio-Demographic Characteristics

Subjects’ age, education and income level, and co-residence were investigated. The researchers classified subjects’ age (40s, 50s, 60s), education level (under elementary-school graduate, middle-school graduate, high-school graduate, or above-college graduate), income level (household income quartile: low, mid-low, mid-high, or high), and co-residence (cohabitation or non-cohabitation).

#### 2.4.2. Lifestyle Features

The researchers investigated subjects’ current smoking, drinking, physical activity, and dietary controls. Answers to the question as to whether the subject currently smokes were classified into “Yes (smoking)” or “No (not smoking).” Answers to the question regarding how frequently the subject drank during the last year were classified into “Yes (drinking)” and “No (not drinking).” Answers to the subject’s physical activity (if they practiced over 2 h 30 min per week of mid-intensity physical activity or over 1 h 15 min per week of high-intensity physical activity or mid-mixed-high-intensity physical activity (1 min of high-intensity physical activity equals 2 min of mid-intensity physical activity)) were classified into “Yes (practiced)” and “No (not practiced)” as the seventh KNHANES‘ guideline by the Korea Centers for Disease Control and Prevention (KCDC) [18]. Regarding dietary control status, answers to the question of whether the subject is currently under dietary control due to special circumstances were classified as “Yes” and “No.” For those who answered “Yes,” the reasons were investigated and reclassified as “Disease,” “Weight control,” or “Others.”

#### 2.4.3. MetS

In this study, those who showed more than three symptoms including abdominal obesity, high blood pressure, hypertriglyceridemia, low HDL cholesterol, and hyperglycemia, were defined as MetS. In addition, these MetS subjects were classified into “3,” “4,” and “5.” Guidelines from the 2005 Adult Treatment Panel III (ATP III) [19], conducted by the American Heart Association (AHA)/The National Heart, Lung, and Blood Institute (NHLBI), were the standard used for MetS diagnosis. In addition, to apply to the Korean situation, the 2014 standard of the Korean Society for Study of Obesity was used for abdominal obesity [20]. Specific standards of MetS diagnosis in this study are as follows:

- Waist circumference: values of ≥85 cm for women
- Blood pressure: systolic pressure ≥130 mmHg or diastolic pressure ≥85 mmHg (or those taking hypertension-related medicines).
- Triglyceride: values of ≥150 mg/dL (or those taking dyslipidemia-related medicines).
- HDL cholesterol: values of ≤50 mg/dL for women (or those taking dyslipidemia-related medicines).
- Fasting blood sugar: values of ≥100 mg/dL (or those taking diabetes-related medicines).

Medicine usage for hypertension, dyslipidemia, or diabetes was investigated regarding current drug treatment status. Furthermore, the subjects’ status was categorized as “Yes (currently taking),” “No, with diagnosis” or “No, without diagnosis.”

### 2.5. Data Analysis

The data were analyzed using SPSS version 24.0 (IBM Corp., Armonk, NY, USA). Differences were considered statistically significant at *p* < *0*.05. Socio-economic status, lifestyle, and MetS status were reported as the average/standard error, frequency, and percentage applying weight. Furthermore, differences in dietary control and physical activity were compared by socio-economic status, lifestyle, and MetS variables, which comprise the number of MetS and each component of MetS, using chi-square test and t-test in complex sample analysis. Lastly, multiple logistic regression in complex sample analysis was performed to identify influencing factors which are grouped by socio-economic status, lifestyle, and MetS status represented by the variable of the number of MetS on dietary control and undertaking physical activity.

## 3. Results

### 3.1. Socio-Demographic Characteristics, Lifestyle Features, and MetS-Related Characteristics of Study Participants

Regarding the subjects’ general characteristics, the average age was 57.0 years; those in their 50 s were most common (50.6%), those in their 60 s totaled 34.9%; and those in their 40 s totaled 9.1%. The highest-ranking education level was high school graduate (34.1% of subjects), then elementary school graduate (29.9%), middle school graduate (21.5%), and college graduate (14.5%). Income levels were similar among the subjects. Mid–high–income levels totaled 28% of subjects, mid–low–income levels totaled 27.6%, and high–income levels totaled 25.7%. The low–income level group was the smallest group, with 18.7% of subjects. Lastly, those with co-habiting members totaled 91.3% and those with none totaled 8.7%.

The lifestyle survey results showed that 60.3% of subjects were current drinkers, 4.1% current smokers, 39.5% physical activity performers, and 36.1% on dietary control. The reason for each subjects’ dietary controls showed similar dispersion: 50.1% due to weight control, 46.7% due to illness, and 3.2% for other reasons.

Regarding MetS-related characteristics, the average accompanying number of MetS-diagnosed components was 3.6. Those with three components totaled 50.6%, the largest group; those with four components totaled 35.0%; and those with five components totaled 14.3%. Among MetS-diagnosed components, subjects’ average waist measurement was 86.0 cm, and 54.9% of those subjects had abdominal obesity. The average systolic pressure was 125.9 mmHg, the average diastolic pressure was 79.3 mmHg, and 69.0% of subjects had high blood pressure. The average triglyceride was 185.6 mg/dL, and 83.0% of subjects had high triglyceride. The average HDL cholesterol was 47.6 mg/dL, and 87.6% of subjects had high HDL cholesterol. Lastly, the average blood sugar level was 113.7 mg/dl, and 69.1% of subjects had high blood sugar.

As for drug-treatment-related characteristics, specifically regarding hypertension treatment, 56.0% of subjects were not taking medicines because they had not been not diagnosed by a doctor; among those having undergone a doctor’s diagnosis, 42.2% were taking medicines, and 1.8% of subjects were not taking medicines. Regarding medicinal dyslipidemia treatment, 47.6% of subjects were not taking medicines because they had not been diagnosed by a doctor; after a doctor’s diagnosis, 47.6% were taking medicines, and 10.5% were not taking medicines. In the case of diabetes mellitus treatment, 78.0% of subjects were not taking medicines because they had not been diagnosed by a doctor; after a doctor’s diagnosis, 21.8% were currently taking medicine, and 0.1% were not taking medicines (Table 1).

### 3.2. Differences in Dietary Control Status According to Sociodemographic, Lifestyle and MetS-Related Characteristics

Dietary control status analysis according to sociodemographic characteristics was significantly different in age (χ^2^ = 346.59, *p* *<* 0.001), education level (χ^2^ = 206.02, *p <* 0.001), income level (χ^2^ = 20.95, *p* = 0.021), and co-residence (χ^2^ = 17.87, *p* = 0.001). The group on dietary control showed an average age of 56.2, significantly lower than the group who are not on dietary control, which was 57.4 years (*t* = 4.53, *p*= 0.034). According to education level, higher percentages of those on dietary control were elementary school graduates (33.0%, 28.3%) and college graduates (17.4%, 12.6%) than those who are not, relatively less so in middle school graduates. As for subjects’ income level, the dietary control group showed higher percentages of people in the “high-income” group (31.5%, 22.1%) and lower percentages in the “low,” “mid-low,” and “mid-high-income” groups. The dietary control group showed a higher percentage of people in the non-cohabitation group regarding the co-residence status (9.5%, 3.3%).

Dietary control status according to healthy lifestyle was significantly different in the current drinking (χ^2^ = 67.59, *p <* 0.001), current smoking (χ^2^ = 19.17, *p* = 0.003) and physical activity groups (χ^2^ = 64.50, *p <* 0.001). There were more current drinkers in the non-dietary control group (62.1%) than in the dietary control group (53.0%) and current smokers showed the same type of result (4.5%, 3.5%). In contrast, there were more physical activity performers in the dietary control group (49.9%) than in the non-dietary control group (33.8%).

Dietary control status according to MetS characteristics was significantly different in the hypertension drug treatment (χ^2^ = 79.87, *p* < 0.001), dyslipidemia drug treatment (χ^2^ = 187.11, *p* < 0.001), diabetes drug treatment (χ^2^ = 226.88, *p* < 0.001) and accompanying number of MetS components groups (χ^2^ = 113.37, *p <* 0.001). In addition, dietary control status according to all MetS diagnosis components was significantly different in the abdominal obesity (χ^2^ = 98.44, *p <* 0.001), high blood pressure (χ^2^ = 18.59, *p* = 0.001), high triglyceride (χ^2^ = 25.87, *p* < 0.001), low HDL cholesterol (χ^2^ = 50.57, *p <* 0.001) and high blood sugar groups (χ^2^ = 64.99, *p <* 0.001). There was more hypertension drug-taking in the dietary control group (49.4%, 38.2%), and the dyslipidemia (50.4%, 37.3%) and diabetes mellitus drug groups (58.4%, 41.6%) showed the same type of result. As for accompanying number of MetS components, the dietary control group showed an average number of 3.7 subjects and the non-dietary control group showed an average number of 3.6 subjects (*t* = 5.94, *p* = 0.015). The group with three MetS components had fewer subjects in the dietary control group (45.1%) than the non-dietary control group (53.6%). Abdominal obesity was shown in 55.0% of subjects in the dietary control and non-dietary control groups. The high blood pressure (70.0%, 68.8%), high triglyceride (84.1%, 82.4%), low HDL cholesterol (89.1%, 86.7%), and high blood sugar groups (75.9%, 65.1%) all showed higher percentages of subjects in the dietary control group than the non-dietary control group. A difference also occurred in the average blood sugar level group (t = 5.20, *p* = 0.023). The dietary control group showed higher blood sugar levels (119.2 mg/dL) than the non-dietary control group (110.7 mg/dL) (Table 1).

### 3.3. Differences in Physical Activity Status According to Sociodemographic, Lifestyle and MetS-Related Characteristics

Physical activity status according to sociodemographic characteristics was significantly different in the age (χ^2^ = 466.94, *p* < 0.001), education level (χ^2^ = 133.73, *p* < 0.001) and income level groups (χ^2^ = 137.56, *p* < 0.001). In the physical activity performing group, the percentage of those in their 40s (9.7%) and 50s (56.5%) was higher than those in their 40s (8.8%) and 50s (55.6%) in the non-performing group. As for education level, there were more subjects in the physical activity performing group (40.7%) with high school grades than in the non-performing group (29.8%). In contrast, the performing group showed lower numbers of subjects for elementary school graduates, middle school graduates, and college graduates than the non-performing group. Regarding income level, the physical activity group showed fewer subject in the “low-income” group (14.3%, 21.6%), and showed higher subject numbers in the “mid-low,” “mid-high,” and “high-income” groups.

Physical activity status according to lifestyle significantly differed in the current drinking group (χ^2^ = 48.53, *p* < 0.001), dietary control status (χ^2^ = 64.50, *p* < 0.001) and reason for dietary control (χ^2^ = 35.57, *p* < 0.001). Current drinkers had lower numbers in the non-physical activity performing group (59.4%) than in the performing group (62.8%). The percentage of those on dietary control was higher in the physical activity performing group (45.5%) than in the non-performing group (30.0%). Regarding the reason for dietary control, the physical activity group (50.6%) showed higher subject numbers for “weight control” purposes than the non-performing group (49.5%).

Lastly, physical activity status according to MetS characteristics significantly differed in hypertension drug treatment (χ^2^ = 143.36, *p* < 0.001), dyslipidemia drug treatment (χ^2^ = 34.29, *p* < 0.001), diabetes mellitus drug treatment (χ^2^ = 53.96, *p* < 0.001) and accompanying number of MetS components groups (χ^2^ = 131.20, *p* < 0.001). In addition, physical activity status according to all MetS components also significantly differed in abdominal obesity (χ^2^ = 32.59, *p* < 0.001), high blood pressure (χ^2^ = 77.10, *p* = 0.001), high triglyceride (χ^2^ = 31.75, *p* < 0.001), low HDL cholesterol (χ^2^ = 65.91, *p* < 0.001) and high blood sugar groups (χ^2^ = 83.97, *p* < 0.001). There were fewer subjects under high blood pressure drug treatments resulting from a doctor’s diagnosis in the physical activity performing group (37.6%, 45.1%). Those under dyslipidemia (41.7%, 42.1%) and diabetes drug treatments (20.3%, 21.3%) showed similar results. As for an accompanying number of MetS components, the group, which had three MetS components had higher numbers in the physical activity group (54.0%) than in the non-performing group (48.5%). In contrast, abdominal obesity had fewer subject numbers in the physical activity performing group (48.6%) than in the non-performing group (59.0%). Furthermore, average waist measurement showed lower numbers in the physical activity performing group (84.7 cm) than in the non-performing group (86.9 cm) (t = 8.00, *p* = 0.005). High blood pressure (68.0%, 69.7%) and high triglyceride groups (81.5%, 84.0%) also showed similar results. However, low HDL cholesterol (88.9%, 86.8%) and high blood sugar groups (71.3%, 67.6%) showed higher subject numbers in the physical activity performing group than in the non-performing group (Table 2).

### 3.4. Associated Factors of Dietary Control

Table 3 presents the odds ratios (OR) for dietary control according to sociodemographic and lifestyle factors, and MetS characteristics (Table 3). The results show that age, physical activity status, hypertension drug treatment status, and diabetes drug treatment status are the most significant lifestyle modification factors for MetS. Subjects in their 40s were on dietary control 3.380 times more than those in their 60s (OR = 3.380, CI = 1.185–6.815). The physical activity performing group was on dietary control 2.238 times more than the non-performing group (OR = 2.238, CI = 1.425–3.516). The group taking drugs after a doctor’s diagnosis of hypertension were on dietary control 1.662 times more than the group not taking drugs due to never having been diagnosed with hypertension (OR = 1.662, CI = 1.035–2.670). The group under drug treatment for a diabetes mellitus diagnosis was on dietary control 2.992 times more than the group not on diabetes drug treatments due to never having been diagnosed with diabetes mellitus (OR = 2.992, CI = 1.801–4.971).

### 3.5. Associated Factors of Physical Activity

Table 3 also presents the ORs for physical activity according to sociodemographic and lifestyle factors, and MetS characteristics. The results show that dietary control status is also one of the most significant lifestyle modification factors. The dietary control group performed physical activity 2.234 times more than the non-dietary-control group (OR = 2.232, CI = 1.418–3.514).

## 4. Discussion

In this current study, we examined compliance with healthy lifestyles of menopausal MetS women in Korea and analyzed associated factors of dietary control and physical activity depending on socio-economic status, lifestyle, and MetS status. There were three major findings.

First, menopausal MetS women in Korea who maintain a healthy lifestyle totaled around 35% of the subjects under study. Most participants did not smoke (95.9%), and less than half of participants adhered to engaging in physical activity (39.5%), no drinking (39.2%), and dietary control (36.1%). Compared with our subjects who did not smoke, MetS prevalence in these menopausal women might relate to menopausal pathology as estrogen deficiency [1,2] rather than smoking [21,22,23]. In this study, rates of engaging in physical activity (39.5%) were lower compared to previous cross-sectional studies of menopausal women by Kara et al. (47%) [24] and Mansikkamäk et al. (50.7%) [25]. In the present study, compared with 36.1% of subjects with dietary control compliance, Cho et al. reported that more than half of study participants (52.3%) keep healthy dietary patterns in menopausal women through dietary patterns based on dietary-intake records [26]. This discrepancy might relate to the analyzed data types such as usual dietary intake records, or the self-administered questionnaire regarding current dietary control. This difference may be due to variations between evaluations of healthy diet consumption by dietary-intake records and subjects’ perceived efforts of dietary control, which demonstrated different adherence to dietary control. Previous studies demonstrated that dietary control and physical activity constitute the core MetS interventions [12,13,14,15]. The adherence to dietary control and physical activity in this study is lower than previous studies, which suggests that effective interventions may be necessary for menopausal women to improve their compliance with dietary control and physical activity.

Second, women who applied dietary control strategies to improve their health were more likely to be in the 40–49 age group (OR: 3.38; CI: 1.25–9.18), engage in physical activity (OR: 2.24;CI: 1.43–3.52) and take hypertension medication (OR: 1.66; CI: 1.39–3.30) or diabetes mellitus medication (OR: 2.99; CI: 1.80–4.97). In the current study, compared to the 60–64 age group, the OR for adherence to dietary control in the 40–49 age group was 3.38-fold higher. These findings are similar to previous studies, which stated that lifestyle modifications increase with age [27] and those in their 40s are more likely to maintain a healthy lifestyle, whereas those in their 60s and older are less likely to maintain a healthy lifestyle [28]. Thus, considering that lifestyle-modification compliance increases with age, it is necessary to develop a strategy based on age to improve healthy diet control. Given our result of the 2.238-fold higher OR for compliance with dietary control in the physical activity group, these findings are similar to previous studies, which state that physical activity affects individuals’ healthy nutritional needs [29] and increased physical activity improves eating habits [30]. In this study, the OR for dietary control adherence in taking hypertension medication or diabetes mellitus medication were significantly higher. Similarly, previous studies reported that perceived health status and perceived disease status such as diagnosed diseases, health examination, and prescribed medications for those diseases improve healthy lifestyle compliance [31,32]. Our findings that taking anti-hypertensive medication or anti-diabetic medication was positively related to dietary control adherence are considered to be the same results as those in previous studies which found that the perceived health status motivates them to practice lifestyle modification such as diet control [31,32]. The result suggests that it is necessary to have regular health examinations and educational consultations regarding MetS symptoms and complications to improve their perceived health status and support dietary control in menopausal MetS women.

Finally, physically active menopausal women with MetS were more likely to also engage in dieting (OR: 2.32; CI: 1.42–3.51). Compared with this result, previous studies reported that food intake and physical activity associate with each other [33,34,35]. As this study only included mid-intensity and high-intensity physical activity, this may limit our understanding of the more detailed associated factors that increase physical-activity adherence. Future studies must explore more detailed data on physical activity, such as exercise frequency and exercise type, for example walking, balanced training, and strength training.

The design of this study had some limitations. First, because of its cross-sectional nature, we provided representative results based on KNHANES, but we could not prove that causal relationships exist between lifestyle factors. Second, there may be information bias due to using self-reported data to analyze associated factors of lifestyle modification. Third, we had limited information on other influencing factors, such as knowledge, motivation, social support, and self-efficacy [36], regarding MetS healthy lifestyles. Therefore, we had to obtain detailed information about dietary control and physical activity. Examining more detailed dietary patterns, including eating habits, frequency of eating alone and dining out, nutrient profile, and whether subjects skip meals, would help us to better suggest associated lifestyle factors to increase dietary control compliance. A prospective design study is necessary to investigate dietary control and physical activity compliance, including the more detailed lifestyle features.

## 5. Conclusions

We found that the associated lifestyle factors associated with dietary control in menopausal women are within the 40–49 age group who are physically active and take hypertension or diabetes mellitus medication. Our study also showed that adherence to physical activity in menopausal women relates to dietary control. These results support the beneficial impact of customized interventions considering age, compliance to lifestyle, perception of disease, and health status. Accordingly, suggestions can be provided to healthcare workers for designing not only individual approaches to lifestyle modification but also comprehensive intervention including dietary control and physical activity for menopausal MetS women. Health-care interventions like dietary control providing additional support to vulnerable MetS women should target those aged 60 or over those who do not take medicines related to hypertension and diabetes mellitus.

## Figures and Tables

**Table 1:** Differences in Dietary Control by Sociodemographic, Lifestyle and metabolic syndrome (MetS) Characteristics.

Variables		Total	Dietary Control		*χ*^2^ or t	*p*
			Yes (*n* = 199)	No (*n* = 364)		
Age	40–49	35 (9.1)	16 (13.2)	18 (6.5)	346.59	<0.001
	50–59	288 (56.0)	102 (54.9)	186 (56.8)		
	60–64	241 (34.9)	81 (31.9)	160 (36.7)		
	Mean ± SD	57.0 ± 0.26	56.2 ± 0.49	57.4 ± 0.29	4.53	0.034
Education level	≤Elementary	195 (29.9)	65 (33.0)	130 (28.3)	206.02	<0.001
	Middle school	122 (21.5)	37 (15.1)	85 (25.2)		
	High school	170 (34.1)	64 (34.5)	106 (34.0)		
	≥College	75 (14.5)	31(17.4)	4.3 (12.6)		
Income level	Low	110 (18.7)	36 (16.0)	74 (20.3)	20.95	0.021
	Mid-lower	164 (27.6)	50 (24.9)	114 (29.2)		
	Mid-upper	153 (28.0)	54 (27.6)	99 (28.4)		
	High	136 (25.7)	58 (31.5)	77 (22.1)		
Co-residence	Yes	503 (91.3)	176 (90.4)	326 (91.7)	17.87	0.001
	No	61 (8.7)	23 (9.6)	38 (8.3)		
Current drinking	Yes	330 (60.8)	109 (58.0)	220 (62.1)	67.59	<0.001
	No	234 (39.2)	90 (42.0)	144 (37.9)		
Current smoking	Yes	23 (4.1)	7 (3.5)	16 (4.5)	19.17	0.003
	No	539 (95.9)	191 (96.5)	347 (95.5)		
physical activity	Yes	207 (39.5)	90 (49.9)	117 (33.8)	64.50	<0.001
	No	357 (60.5)	109 (50.1)	247 (66.2)		
Hypertension drug	Yes	258 (42.2)	103 (49.4)	155 (38.2)	79.87	<0.001
	No w/diagnosis	9 (1.8)	6 (2.9)	3 (1.2)		
	No w/o diagnosis	297 (56.0)	90 (42.7)	206 (60.6)		
Dyslipidemia drug	Yes	248 (41.9)	99 (50.4)	149 (37.3)	187.11	<0.001
	No w/diagnosis	58 (10.5)	20 (10.5)	38 (10.5)		
	No w/o diagnosis	258 (47.6)	80 (39.1)	177 (52.2)		
Diabetes Mellitus drug	Yes	118 (21.8)	66 (58.4)	52 (41.6)	226.88	<0.001
	No w/diagnosis	1 (0.1)	1 (0.0)	0 (0.0)		
	No w/o diagnosis	445 (78.0)	132 (29.8)	312 (70.2)		
Number of MetS components	3	281 (50.6)	90 (45.1)	190 (53.6)	113.37	<0.001
	4	193 (35.0)	67 (35.6)	126 (34.8)		
	5	90 (14.3)	42 (19.3)	48 (11.6)		
	Mean ± SD	3.6 ± 0.03	3.7 ± 0.06	3.6 ± 0.04	5.94	0.015
Waist Circumstance	Normal	247(45.1)	85 (45.0)	161 (45.0)	98.44	<0.001
	Abnormal	317 (54.9)	114 (55.0)	203 (55.0)		
	Mean ± SD	86.0 ± 0.47	86.1 ± 0.78	86.1 ± 0.58	0.00	0.999
Blood Pressure	Normal	151 (31.0)	53 (30.0)	97 (31.2)	18.59	0.001
	Abnormal	413 (69.0)	146 (70.0)	267 (68.8)		
	Mean ± SD	125.9 ± 0.69/79.3 ± 0.44	124.0 ± 1.31/78.2 ± 0.68	127.1 ± 0.89/79.9 ± 0.57	3.443.37	0.0640.067
TG	Normal	103 (17.0)	35 (15.9)	68 (17.6)	25.87	<0.001
	Abnormal	461 (83.0)	164 (84.1)	296 (82.4)		
	Mean ± SD	185.6 ± 7.65	169.4 ± 10.84	189.9 ± 8.71	2.21	0.137
HDL-C	Normal	69 (12.4)	24 (10.9)	45 (13.3)	50.57	<0.001
	Abnormal	495 (87.6)	175 (89.1)	319 (86.7)		
	Mean ± SD	47.6 ± 0.56	47.5 ± 0.90	47.7 ± 0.68	0.06	0.809
Blood glucose	Normal	185 (30.9)	50 (24.1)	135 (34.9)	64.99	<0.001
	Abnormal	379 (69.1)	149 (75.9)	229 (65.1)		
	Mean ± SD	113.7 ± 1.76	119.2 ± 3.36	110.7 ± 1.82	5.20	0.023

Mean ± SD: Mean ± standard daviation; TG: Triglyceride; HDL-C: HDL cholesterol.

**Table 2:** Differences in Physical Activity by Sociodemographic, Lifestyle and MetS Characteristics.

Variables		Total	Physical Activity		*χ*^2^ or t	*p*
			Yes (*n* = 207)	No (*n* = 357)		
Age	40–49	35 (9.1)	12 (9.7)	23 (8.8)	466.94	<0.001
	50–59	288 (56.0)	109 (56.5)	179 (55.6)		
	60–64	241 (34.9)	86 (33.8)	155 (35.6)		
	Mean ± SD	57.0 ± 0.26	56.9 ± 0.48	57.0 ± 0.32	0.09	0.768
Education level	≤Elementary	195 (29.9)	61 (25.5)	134 (32.8)	133.73	<0.001
	Middle school	122 (21.5)	46 (21.2)	76 (21.7)		
	High school	170 (34.1)	71 (40.7)	99 (29.8)		
	≥College	75 (14.5)	28 (12.7)	47 (15.7)		
Income level	Low	110 (18.7)	29 (14.3)	81 (21.6)	137.56	<0.001
	Mid-lower	164 (27.6)	67 (29.3)	97 (26.5)		
	Mid-upper	153 (28.0)	58 (30.3)	95 (26.5)		
	High	136 (25.7)	53 (26.2)	83 (25.3)		
Co-residence	Yes	503 (91.3)	189 (93.3)	314 (89.9)	5.93	0.071
	No	61 (8.7)	18 (6.7)	43 (10.1)		
Current drinking	Yes	330 (60.8)	130 (62.8)	200 (59.4)	48.53	<0.001
	No	234 (39.2)	77 (37.2)	157 (40.6)		
Current smoking	Yes	23 (4.1)	5 (2.8)	18 (5.0)	3.01	0.203
	No	539 (95.9)	201 (97.2)	338 (95.0)		
Dietary Control	Yes	199 (36.1)	90 (45.5)	109 (30.0)	64.50	<0.001
	No	364 (63.9)	117 (54.5)	247 (70.0)		
Reason of Dietary Control	Disease	89 (46.7)	40 (46.7)	49 (46.6)	35.57	<0.001
	Weight control	104 (50.1)	48 (50.6)	56 (49.5)		
	Others	6 (3.2)	2 (2.6)	4 (3.8)		
Hypertension drug	Yes	258 (42.2)	85 (37.6)	173 (45.1)	143.36	<0.001
	No w/diagnosis	9 (1.8)	5 (3.3)	4 (0.8)		
	No w/o diagnosis	297 (56.0)	117 (59.1)	180 (54.0)		
Dyslipidemia drug	Yes	248 (41.9)	88 (41.7)	160 (42.1)	34.29	<0.001
	No w/diagnosis	58 (10.5)	20 (10.6)	38 (10.3)		
	No w/o diagnosis	258 (47.6)	99 (47.7)	159 (47.6)		
Diabetes Mellitus drug	Yes	118 (21.8)	42 (20.3)	76 (21.3)	53.96	<0.001
	No w/diagnosis	1 (0.1)	1 (0.5)	0 (0.0)		
	No w/o diagnosis	445 (78.0)	164 (79.2)	281 (78.7)		
Number of MetS components	3	281 (50.6)	110 (54.0)	171 (48.5)	131.20	<0.001
	4	193 (35.0)	70 (33.6)	123 (35.9)		
	5	90 (14.3)	27 (12.4)	63 (15.6)		
	Mean ± SD	3.6 ± 0.03	3.6 ± 0.06	3.7 ± 0.05	2.78	0.096
Waist Circumstance	Normal	247 (45.1)	101 (51.4)	146 (41.0)	32.59	<0.001
	Abnormal	317 (54.9)	106 (48.6)	211 (59.0)		
	Mean ± SD	86.0 ± 0.47	84.7 ± 0.64	86.9 ± 0.58	8.00	0.005
Blood Pressure	Normal	151 (31.0)	58 (32.0)	93 (30.3)	77.10	<0.001
	Abnormal	413 (69.0)	149 (68.0)	264 (69.7)		
	Mean ± SD	125.9 ± 0.69/79.3 ± 0.44	126.0 ± 1.16/79.7 ± 0.74	125.9 ± 1.06/79.0 ± 0.63	0.010.53	0.9280.468
TG	Normal	103 (17.0)	42 (18.5)	61 (16.0)	31.75	<0.001
	Abnormal	461 (83.0)	165 (81.5)	296 (84.0)		
	Mean ± SD	185.6 ± 7.65	176.1 ± 11.01	191.8 ± 10.04	1.16	0.681
HDL-C	Normal	69 (12.4)	25 (11.1)	44 (13.2)	65.91	<0.001
	Abnormal	495 (87.6)	182 (88.9)	313 (86.8)		
	Mean ± SD	47.6 ± 0.56	47.6 ± 0.84	47.6 ± 0.71	0.00	0.968
Blood glucose	Normal	185 (30.9)	64 (28.7)	121 (32.4)	83.97	<0.001
	Abnormal	379 (69.1)	143 (71.3)	236 (67.6)		
	Mean ± SD	113.7 ± 1.76	113.0 ± 2.77	114.2 ± 2.16	0.11	0.742

**Table 3:** Associated Factors of Dietary Control and Physical Activity.

Variables		Dietary Control			Physical Activity		
		OR	95% CI	*p*	OR	95% CI	*p*
Age	40–49	3.380	1.245–9.178	0.017	0.662	0.279–1.571	0.348
	50–59	1.229	0.769–1.966	0.388	0.871	0.560–1.354	0.540
	60–64	1.000			1.000		
Education level	≤Elementary	1.302	0.626–2.709	0.479	0.964	0.454–2.049	0.924
	Middle school	0.517	0.256–1.042	0.065	1.328	0.622–2.835	0.463
	High school	0.741	0.379–1.447	0.379	1.882	0.914–3.875	0.086
	≥College	1.000			1.000		
Income level	Low	0.513	0.258–1.023	0.058	0.799	0.388–1.647	0.543
	Mid-lower	0.529	0.278–1.008	0.053	1.318	0.733–2.371	0.355
	Mid-upper	0.627	0.339–1.159	0.136	1.067	0.612–1.859	0.820
	High	1.000			1.000		
Co-residence	No	1.521	0.812–2.848	0.190	0.696	0.326–1.489	0.350
	Yes	1.000			1.000		
Current drinking	Yes	0.645	0.415–1.004	0.052	1.236	0.798–1.913	0.342
	No	1.000			1.000		
Current smoking	Yes	0.769	0.244–2.426	0.654	0619	0.194–1.977	0.418
	No	1.000			1.000		
physical activity	Yes	2.238	1.425–3.516	<0.001	-	-	-
	No	1.000			-	-	-
Dietary control	Yes	-	-	-	2.232	1.418–3.514	0.001
	No	-	-	-	1.000		
Hypertension drug	Yes	1.662	1.035–2.670	0.036	0.699	0.442–1.107	0.127
	No w/diagnosis	3.401	0.655–17.666	0.145	2.955	0.611–14.033	0.178
	No w/o diagnosis	1.000			1.000		
Dyslipidemia drug	Yes	1.504	0.932–2.427	0.094	0.916	0.585–1.433	0.700
	No w/diagnosis	1.433	0.687–2.987	0.337	0.935	0.477–1.830	0.844
	No w/o diagnosis	1.000			1.000		
Diabetes Mellitus drug	Yes	2.992	1.801–4.971	<0.001	1.150	0.676–1.956	0.065
	No w/diagnosis *						
	No w/o diagnosis	1.000			1.000		1.000
Number of MetS components	3	0.749	0.384–1.459	0.395	1.399	0.694–2.820	0.348
	4	0.632	0.322–1.238	0.181	1.199	0.607–2.369	0.601
	5	1.000			1.000		

* This group was excluded from the multiple logistic regression analysis because the subject number of this group was ‘1′ only.
